# Supplementary material for: Are carbon emissions trading and green financial instruments synergistic? -Comprehensive quantitative research based on content analysis
Source: PLoS One. 2024 Mar 7;19(3):e0298601. doi: 10.1371/journal.pone.0298601 (PMC10919601; doi:10.1371/journal.pone.0298601)
Supplement: S1 Data — (ZIP) [file pone.0298601.s001.zip › Data/地级市Z_F工作报告.zip!/║┌┴·╜¡/╞▀╠¿║╙╩╨/└┤╫╘╣1⁄2╓┌║┼í╛┬φ┐╦╩2╛▌═°í┐_2008.docx]

各位代表：

　　现在，我代表七台河市人民政府，向大会作政府工作报告，请予审议，并请列席会议的同志提出意见。

　　一、2008年工作回顾

　　2008 年，是我市开创跳跃式发展新局面的一年。一年来，国内外经济环境发生了复杂深刻的变化，面对日益严峻的形势，在省委、省政府和市委的正确领导下，在市人大、市政协的监督和支持下，我们紧紧依靠和带领全市人民，深入学习实践科学发展观，积极应对各种困难和挑战，凝心聚力加快发展，千方百计改善民生，圆满完成了市九届人大三次会议确定的各项任务。

　　这一年，是经济增长明显加快的一年。预计地区生产总值实现187 亿元，增长26%，人均GDP 突破2 万元。全口径财政收入32.5 亿元，增长72.2%。社会消费品零售总额36 亿元，增长20%。外贸进出口总额3500 万美元，增长26%。6 项指标增幅居全省前列，7 项指标创历史最好水平。

　　这一年，是发展能力明显增强的一年。工业固定资产投资完成70 亿元，增长52.2%。开工建设千万元以上项目86 个，其中亿元项目17个。一批大项目建成投产，开始了新一轮产业扩能。辟建金河工业集中区，完成了一期配套工程、创业中心主体工程，奠定了七台河未来发展的广阔空间。

　　这一年，是群众得到实惠明显增多的一年。城镇居民人均可支配收入和农民人均纯收入分别增长15.8%和16%。城乡居民居住条件有效改善。用于民生的各类补贴3.1 亿元，办了一大批群众热切盼望的大事、实事。

　　这一年，是城乡面貌明显变化的一年。完成七桦路、湖滨广场、城市南北出口景观带等工程建设。“十万绿树进城区、十万绿树进矿山”成果显著。拆除了以桃山水库北岸建筑为重点的各类违章建筑472 处，撤销了七桦收费站。开工建设医疗垃圾处理场、污水处理厂。关闭小煤矿、淘汰落后产能，城乡面貌及环境质量大幅提升。

　　这一年，是喜事连连、捷报频传的一年。市政府蝉联全国扶持中小企业创新发展成绩显著地方政府。成功创办了首届家具节暨家居文化博览会，矿区开发建设50 周年、世界大冬会火炬传递、中国乒超联赛等活动规模空前。6 项专利获国际发明展览会大奖，5 户企业被评为国家或省名牌产品企业，获全国冬运会奖牌榜第四名，七台河的城市品牌形象和影响力都有了较大提升。

　　这些成就，标志着我市在“发展循环经济，建设龙江强市”的进程中又迈出坚实的一步，进一步增强了全市人民继续前进的信心和勇气。

　　一年来的主要工作是：

　　（一）提高工业运行质量，扩大工业产出能力。以“循环经济推进年”为载体，全力培育工业竞争优势。规模以上工业增加值实现93.4 亿元，同比增长48%，销售收入、利税分别达到255 亿元、35 亿元，分别增长93.2%、90.2%。煤炭、焦化、电力等主导产业快速发展。煤化工产业居全省前列，宝泰隆、凯博达98 万吨焦炉项目开工建设，吉伟焦炉、兴盛和万昌苯加氢、宝泰隆甲醇、大唐两台60 万千瓦机组等一批大项目建成投产。以木制品加工为代表的接续产业不断壮大，配套企业发展迅猛。

　　（二）强化“三农”工作，扎实推进新农村建设。针对春旱威胁、农资涨价等突出问题，综合采取多种措施，实现一次播种保全苗，粮食产量达到12 亿斤，创历史新高。加快发展特色农业和畜牧业，新增棚室238 栋、养殖小区5 个。转移农村劳动力4.5 万人、创收2.7 亿元。投入新农村建设资金1.7 亿元，建成农村公路305 公里，解决15 个村屯、近万人饮水安全问题，新建沼气池1274 个，18个省级试点村全部达标。农村各项事业全面发展，农民享受的公共服务日益增多，农村面貌发生了可喜变化。

　　（三）加快改革开放步伐，增强经济发展活力。建设“负责任政府”，在全省率先完成解欠国企职工工资任务，受到省领导贺电表扬。市电业局晋升为省直属局。扎实推进“抓大扶小”工程，全市纳税超千万元民营企业达到28 户、新增11户，其中纳税超亿元企业4 户、新增2 户，非公经济占全市经济总量首次超过60%，成为安置就业的主体，壮大财源的生力军，推进经济加快发展的增长点。第三产业税收增长首次突破50%。对上争取项目75 个，到位资金8 亿元。对外贸易努力克服金融危机带来的冲击，实现了速度和效益同步增长。首届家具节有俄、日、韩等国代表团及国内外166 户企业参展，参观人数超过15 万人次。加强了与友城阿尔乔姆市的高层互访，密切了友好往来。组织参加了莫斯科食品节、哈洽会等一系列经贸活动。全力推进招商引资，严格激励约束机制，引进资金103 亿元。

　　（四）全面开展环境治理，努力改善城乡面貌。不断完善城市规划，提升城市功能。开展“城乡环境治理年”活动，完成一批基础设施项目建设。进一步提高城市管理水平，加强土地管理和利用工作。“学习阳泉经验、推动科学发展、创建山水园林城市”全面启动。进一步加强生态环境建设和污染治理，实施重点减排项目27 个，淘汰落后产能67 万吨，全面完成节能减排任务。加快城市水、气、热等工程和农村“山水林田路、庭院圈厕畜”改造。

　　（五）大力发展社会事业，努力保障和改善民生。全部免除城乡义务教育阶段学杂费，城乡困难家庭学生救助实现全覆盖。完成了三所学校1.2 万平方米校舍建设。大力改善农村办学条件，扎实推进“双高普九”。技师学院、宝泰隆煤化工博士后科研工作站获得正式批复。健全公共卫生服务体系，建设社区卫生服务中心，改扩建乡镇卫生院5 所，加快农村标准化卫生所改造。科技、广播电视、文化、体育全面发展，老干部和青少年活动中心如期竣工，流浪儿童救助保护中心完成主体工程。沉陷区治理新建住宅22 万平方米，启动棚户区改造，改造农村泥草房2800 户，首次新建廉租房、经济适用房570 套、货币补贴1842 户。为下岗失业人员减免税费、发放小额贷款2389 万元，兑现灵活就业人员社保补贴1355 万元，新增就业1.3 万人。城镇居民医疗保险全面启动，参保率达72%，新农合参合率提高到95.9%。失业保险、医保、低保、五保户供养、优抚对象待遇标准均有较大幅度提高，为城镇低保对象发放供热、电价、物价各类补贴2954 万元。

　　（六）切实加强安全生产，全面推进平安建设。扎实开展安全生产百日攻坚战、安全隐患整改年活动，以煤矿安全生产为重点，严格落实责任制，严厉查处事故责任人，全面做好各个领域的隐患排查和专项治理工作，各类事故起数、死亡人数分别下降54.8%、24.7%。连续27 年无森林火灾。加强“三基”工程建设，以奥运等重大活动安保为重点，深入开展社会治安综合治理，群众安全感普遍增强。畅通群众诉求渠道，认真开展领导干部大接访活动，集中解决了一批疑难信访案件，信访工作呈现“三降三无”良好态势。

　　（七）加强民主法制建设，进一步转变政府职能。认真执行市人大及其常委会的决议、决定，自觉接受市人大常委会依法监督，主动接受市政协民主监督，办理人大议案、建议和政协提案176件。完善政府议事规则和程序。强化行政执法监察和效能监察，严格落实行政执法责任制和行政问责制。全面开展解放思想大讨论，扎实开展服务型政府建设年活动，政务服务中心和社区便民中心建成使用。积极推进政务公开，政府工作透明度和公共信息服务水平得到提高。

　　（八）积极筹集资金，帮助灾区恢复重建。四川汶川发生特大地震后，全市人民弘扬“一方有难，八方支援”的抗震救灾精神，共捐赠款物近2000 万元，各级财政支援剑阁县资金859 万元。及时派出公安交警、建筑专家及医疗卫生三批人员赶赴第一线开展抢险救灾和对口援建。积极推进援藏项目建设，实现了我市和西藏日喀则地区高层互访。投资1500 万元，完成援建仁布县4 个项目。

　　过去一年，人事、统计、审计、外事、侨务、老龄、气象、地震、地方志、档案、民兵、预备役等工作得到加强，民族宗教、新闻出版、人口计生、妇女儿童、残疾人等工作也取得长足进步。

　　各位代表，在各种不利因素明显增多、区域经济竞争加剧、财力十分紧张的情况下，我们集中财力、物力和人力，办成了一些多年想办而没有条件办的大事和实事，解决了一些多年想解决而没有能力解决的突出问题，走在了全省经济社会发展的前列。这些成绩是市委统揽全局、正确领导的结果，是市人大、市政协监督支持的结果，是全市人民共同奋斗的结果。在此，我代表市人民政府，向全市各族人民，向所有关心、支持七台河发展的各界人士，表示诚挚的感谢！

　　总结一年来的工作，我们也清醒地认识到，经济社会发展和政府工作还存在许多问题：

　　——保持经济持续快速增长的压力大。受经济大环境变化影响，自去年9 月份开始我市部分经济指标增速逐步回落，焦炭市场需求下降、价格回落；就业再就业形势严峻。

　　——长期形成的结构性矛盾依然比较突出。产业结构调整任务艰巨，农业抵御自然灾害能力弱，工业经济增长的资源环境代价大，现代服务业比重小，扩大出口、利用外资难度大；人才资源匮乏，科技成果转化能力有待提高。

　　——安全生产形势严峻。公共安全事故、灾害事故隐患较多，城市基础设施承载能力相当脆弱，突发事件应急处理能力需要进一步增强，全社会安全防范和自救能力较弱；节能减排、环境保护形势不容乐观。

　　——社会管理和公共服务水平有待进一步提高。公共财力较弱，公共产品和服务供给不足，基础建设欠账大，保障和改善民生任务重，部分群众困难还没有从根本上得到解决；因拆迁、征地、调价引发的上访问题依然比较集中。

　　——政府自身建设需要加强。部分领导干部思想不够解放；一些工作人员责任意识和服务意识不强，不作为、乱作为、中梗阻问题依然存在，改善发展环境的任务依然紧迫；政风建设、廉政工作还需要进一步加强。

　　我们必须以对国家和人民高度负责的精神，采取更加有力的措施，切实解决这些问题，决不辜负人民的期望。

　　二、2009年工作安排

　　2009 年，可能是进入新世纪以来我市经济发展最为困难的一年，同时也是充满机遇而富于挑战的一年。国际金融危机尚未见底，对实体经济的影响进一步加深，对我市经济的负面影响正在逐步扩大和蔓延，其严重后果还会进一步显现，我市发展的外部环境将更趋复杂、更为严峻，经济增长的压力前所未有。但我市经济发展的基本面和快速增长的态势没有因此而逆转，我们面临的困难仍然是前进中的困难，是暂时的、可以克服的。一是经济高速增长奠定了相当的物质基础，具有一定的抵御风险能力；二是世界能源紧缺的大格局不会改变，煤炭行业不可能长期低迷；三是我市产业结构处于上游且符合国家产业政策，在经济发展大局中的基础地位不会改变；四是国家和省实施应对危机、确保增长的一系列政策，为我市经济发展带来了前所未有的重大机遇。我们一定要深刻认识这场危机的严峻性、持续性和不确定性，切实增强危机感和紧迫感，把困难估计得充分些，把措施谋划得周密些，注重从变化的形势中捕捉和把握难得的发展机遇，在逆境中发现和培育有利因素，坚定信心，扎实工作，努力化“危”为“机”，走出一条调整时期经济社会加快发展的新路子，把七台河的改革发展不断推向前进。

　　政府工作的基本思路和任务是：以邓小平理论和“三个代表”重要思想为指导，全面贯彻党的十七届三中全会、省委十届六次全会、市委七届五次全会精神，深入落实科学发展观，全面落实省委“建设八大经济区、实施十大工程”战略部署，围绕“发展循环经济、建设龙江强市”，立足扩大内需保持经济平稳较快增长，加快发展方式转变和结构调整提高可持续发展能力，推进改革开放增强经济发展活力和动力，加快社会建设保障和改善民生，坚持依法行政促进社会和谐稳定，奋力开创经济社会跳跃式发展新局面，以优异成绩迎接新中国成立60 周年。

　　综合各种因素，今年国民经济和社会发展的主要目标是：在优化结构、提高效益和降低消耗、保护环境的基础上，地区生产总值确保增长15%、力争达到20%，财政一般预算收入增长15%，全社会固定资产投资确保增长30%、力争达到全省平均水平，城镇居民人均可支配收入确保增长13%、力争达到16%，农民人均纯收入确保增长11%、力争达到14%，万元GDP 能耗下降6.5%，城镇登记失业率控制在4.5%以内。上

　　述指标的确定，充分考虑了金融危机的负面影响和宏观调控政策的积极因素，既体现积极进取、自我加压精神，又是十分审慎、留有余地的。这仅是一个预期性、指导性指标，最终实现情况与预期目标可能会有一定差距。但如果大环境平稳，我们应对得力，执行的结果会更好一些。

　　今年是实施“十一五”规划和“358”跳跃式发展规划的攻坚之年，保持经济平稳较快增长，具有特别重大的意义。要把握好以下原则：一是力促经济增长。把保持经济平稳较快增长作为经济工作首要任务，抓住重点行业、重点产业和重点企业，加大项目投资，大力培育新的经济增长点。二是力促结构调整。推进产业结构优化升级，加强资源节约和环境保护。做强传统产业，提高市场竞争力；做大新兴产业，增强可持续发展能力。三是力促改善民生。顺应人民群众求富裕、求公正、求平安的愿望，把解决群众关心的热点难点问题作为政府工作的重中之重，维护社会公平正义，确保大部分财力投向民生、投向社会公共事业。四是力促改革开放。创新发展理念，完善发展思路，改善发展环境，敢于突破惯例，破解发展难题，进一步扩大对外开放的广度和深度。

　　要着力抓好以下八个方面的工作：

　　（一）坚定不移地实施投资拉动和项目建设。抓投资既是应对危机保增长的关键之举，更是推动经济社会发展的持久动力。当前，我们正面临抓投资、上项目十分难得的大气候大环境，要放心、放胆、放手地扩大投资，进一步优化投资结构，扩大投资主体，突出投资重点，加强投资管理。加大向上争取力度，高标准谋划并尽早启动农林水利、基础设施、工业交通等方面的68 个大项目。

　　强力推进招商引资，力争引进超千万元项目80个，到位资金105 亿元。在公路建设上，计划投资35.2 亿元，开工建设依七高速公路。投资3 亿元，改扩建七勃公路。扩建鹤大高速七台河至鸡西、佳木斯至牡丹江段。改造扩建城区道路。加快通村公路建设，力争年底前行政村全部通硬化路。在铁路建设上，七密铁路前期有实质性进展。完成新火车站选址、设计等前期工作。在水利建设上，加快灌区节水改造工程建设。续建桃山水库二期工程。开工建设城市第二水源地。对病险水库进行消险加固。在城市基础设施建设上，高标准开工建设体育会展中心。完成污水处理厂、新兴区垃圾处理场建设。同时，要进一步加强与中省直企业合作共建。

　　（二）坚定不移地推动产业结构优化升级。充分发挥优势，挖掘潜力，全面实施《循环经济发展规划》，努力稳定一产、做强二产、做大三产，特别是要集中力量做大做强工业，推进新型工业化进程。

　　稳步发展壮大主导产业。原煤采掘业要加大向上争取、勘探力度，尽快解决煤炭接续资源问题。在确保安全前提下，煤炭产量稳定在1800 万吨。通过新建、改扩建、整合以及改造生产工艺等措施，提高矿井单产能力。焦炭产业要重点谋划工艺设施先进、技术含量高、产品竞争力强的百万吨以上焦炉和节能减排改造项目，通过“关小建大”，提高产业整体水平，力争全市焦炭产能登上800 万吨台阶。煤化工产业要进一步扩大规模，增加品种，延长产业链，重点谋划大型二甲醚、醋酸、乙醇等精细化工项目，保持在全省的排头兵地位。电力产业要积极协调解决电力输出瓶颈问题，加快谋划和建设余热、风力、瓦斯、矸石发电项目，使我市尽快成为东部综合电力基地。支持企业技术改造和技术创新，提高管理水平，鼓励企业强强联合，提高竞争力和抗风险能力。

　　扶强壮大优势产业。依托现有产业基础和优势，通过利益联结、投资引导、政策优惠、行政协调等办法，引导木制品、农机制造、农副产品加工、新型建材等具有比较优势的产业，提高产品科技含量，降低生产成本，延长产业链条，形成聚集效应，扩大规模，占领市场，形成新的竞争优势，增强可持续发展能力。

　　积极培育发展新兴产业。认真落实促进服务业加快发展的各项政策，大力发展旅游、物流、会展等前景好、污染小的现代服务业。加快重点旅游景区及配套建设，加强旅游公共基础设施建设，扩大宣传，强化管理，打造品牌。开辟我市到俄罗斯图里洛格、乌苏里斯克客货运输线路。改造提升传统服务业，发展壮大一批特色商业街和专业市场。大力发展现代服务业，加快物流园区和现代物流网络体系建设，培育扶持物流龙头企业；高度重视并推动金融业加快发展；加快引进和培育服务外包企业；办好以第二届家具节为重点的各种会展和体育赛事；规范发展会计、审计、公证、资产评估、信用评级、招投标等中介服务业。有序发展房地产业，积极发展商业地产和工业地产；推动一、二级市场和租赁市场健康发展，满足不同收入群体的多层次需求。

　　加快培育新的经济增长点。积极发展高新技术产业、医药产业，整合科技资源，加快科技成果推广和产业化步伐。鼓励中小企业“攀高结贵”、“引联挂靠”。高度重视人才，加强对各级各类人才的培养、引进和使用。

　　（三）坚定不移地加快城乡一体化建设。以推进城乡一体化为突破口，以城乡一体化建设推进年为载体，坚持用产业化提升农业、城镇化带动农村、工业化致富农民，扎实推进新农村建设，促进农业增效、农民增收、农村环境全面改善，率先在全省破除城乡二元结构，率先在全省形成城乡经济社会发展一体化新格局。

　　统筹土地利用和城乡规划。抓紧编制完成城乡一体化总体规划，科学制定县区城镇建设、农田保护、产业集聚、村落分布、生态涵养等子规划。坚决维护规划的权威性，分步实施，确保全面落实。

　　统筹城乡产业发展。创新矿区、林区、垦区和城乡合作共建机制，加快县域工业化、城镇化、现代化步伐。优化种植业结构，大力发展特色农业、设施农业。大力发展畜牧业，新增养殖小区20个、专业户100 户。推进农业产业化经营，壮大水稻、大豆、玉米、蔬菜、烤烟五条农产品加工产业链，带动基地面积150 万亩以上。培育肉鸡、生猪、大鹅三条畜产品加工产业链。大力发展农民专业合作组织，农机合作社突破20 个。加快发展农村二、三产业，引导各类生产要素在城乡间合理有序流动。

　　统筹城乡基础设施建设和公共服务。加快发展农村公共事业，完成20 个村屯饮水安全工程，推广农村新型能源。加快农村寄宿学校建设，改善农村办学条件。新建扩建2 个乡镇卫生院和5个乡镇计生服务站，推进145 所村卫生所和计生服务室标准化建设。整合利用乡村办公场所，加强文化设施建设。健全农村社会保障体系，提高新农合参合率，做好五保户供养、受灾群众救助、困难户和残疾人帮扶工作。

　　统筹城乡劳动就业。鼓励企业吸纳本地农民就业。扶持进城农民自主创业。加强就业能力培训，引导农民有序外出就业。努力改善农民进城就业和返乡创业环境。鼓励城市下岗职工、失业人员到农村就业。保护农民工权益，逐步实现农民工劳动报酬、子女就学等与城镇居民享有同等待遇。

　　统筹城乡社会管理。加快农村城市化进程，推动农民市民化，打破城市与乡村的体制分治，改革城乡分割的户籍管理制度，缩小并逐步消除农民与城市居民之间的权利差别，让更多的农村居民享受城市文明。推进小城镇建设和城中村改造试点工作，加快农民向小城镇集聚进程。

　　（四）坚定不移地推进山水园林城市建设。围绕创建国家园林城市目标，扎实开展山水园林城市建设年活动。

　　强规划控制和引导。进一步完善城市规划区控详规划、全市土地总体利用规划。完成金河工业集中区域控详规划。开展城市重点问题规划研究，加强乡镇、村屯规划编制。合理有序拓展城市空间，全面提升城市品位和形象。加强城乡规划管理，强化节约集约用地。

　　加强基础设施建设。扩建改造七勃公路，完成仙洞山环山路机动车道建设。改造城区路，综合治理重点交通节点。做好新火车站、七密铁路项目前期工作。

　　加快城乡绿化步伐。新区要同步推进绿化建设，老区要见缝插绿，千方百计增加绿量。开工建设城市中心广场，高标准搞好仙洞山风景区、城市出口荷花园、学府街东延地段等园林绿化工程，新增城市绿地480 亩，逐步形成一街一景的景观效果。大力开展村屯绿化，完成211 个村屯6500 亩的绿化造林任务。全面开展公路和铁路沿线、小区、庭院、厂矿、库区、河渠沿岸绿化，完成造林绿化4 万亩。

　　加快“三供四治”工程建设。供水重点加快管网改造，新建改造8 条管线。供热重点推进项目建设，完成3 个居民区和金河工业集中区域管网建设。供气重点推进燃气工程建设，抓好煤气供应焦炉、调压站和储气柜建设，新增煤气用户6000户。完成市污水处理厂建设，形成较为完整的排污系统。完成新兴区垃圾处理场和三区垃圾中转站建设。整治服务业原煤散烧和工业企业超标排放、矸石山自燃和煤灰等粉尘污染。提高矸石综合利用水平，对城市主干道和中心区视野内裸露山体进行培土造绿，加快矸石山减量，启动倭肯河治理、桃山水库坝下裸露山体治理工程。

　　切实抓好节能减排和生态建设。严格新建项目“三同时”管理，严把新上项目准入和验收关。淘汰落后产能，做好建筑节能推广工作。扎实推进松花江流域水污染防治项目，加强水源地生态保护。加大重点行业、重点企业的监管力度，特别要严格督促焦化和电力企业上齐节能减排设施，确保污染防治设施稳定达标运行。加快发展循环经济产业链。

　　大力推进城乡环境整治。实施精细化管理，城区要以综合整治“乱停乱行、私接乱建、乱倒乱扔、乱堆乱放、乱贴乱画”为重点，加大依法管理力度，建立市容管理长效机制。加大城市绿化、美化、亮化工作力度。合理划分、严格规范市区两级权限，向基层放权力、给条件、落责任，发挥各区在城市管理中的作用。农村要以综合整治“柴草乱垛、粪土乱堆、垃圾乱倒、污水乱排、畜禽乱放”为重点，开展经常性的清除垃圾、打通道路、清理边沟、改造厕所等工作，推广沼气、秸秆燃气炉等清洁能源。

　　（五）坚定不移地推进改革开放。坚持社会主义市场经济的改革方向，抓住影响和制约发展的关键环节，完善有利于实现保增长、扩内需、调结构的体制机制。

　　抓好重点领域改革。继续深化农村综合改革，坚持家庭联产承包经营基本制度不动摇，积极稳妥地推进土地承包经营权流转，发展多种形式的适度规模经营。加快集体林权制度改革。推进公共财政管理体制改革，深化预算制度改革。深化金融体制改革，改善金融结构和服务。深化价格体制改革，理顺价格关系。推进投资体制改革，健全政府投资管理体制。认真做好行政管理体制改革的有关工作，积极稳妥地推进政府机构改革。

　　继续毫不动摇地发展非公有制经济。毫不动摇地鼓励、支持和引导非公有制经济发展，鼓励全民创业。突出抓好中小企业成长工程，发挥好中小企业发展专项资金的引导作用。发挥好中小企业创业孵化基地作用，鼓励中小企业围绕主导产业和大企业进行协作配套。

　　积极推进全方位对外开放。抓住国家支持出口基地建设机遇，加快建设以农产品加工等优势产业为主导的对俄出口加工基地。培育壮大一批重点外贸企业。鼓励企业“走出去”，引导企业到俄罗斯阿尔乔姆市兴办制砖、木材加工、保温材料等企业；鼓励双叶公司扩大国际市场份额。加强与俄、日、韩等国交流与合作。尽快开通到俄罗斯滨海边区客货运输线路。抓住人民币升值机遇，在稳定出口的同时，扩大关键设备和先进技术的引进。

　　全力抓好招商引资。依托资源优势，做好资源勘探、煤化工、农副产品加工、物流、旅游等招商项目谋划和包装，着力引进一批立市企业。积极做好承接发达地区产业转移招商。依托各类园区、创业中心和孵化器等载体招商。调动社会各界参与招商引资的积极性，加大项目跟踪服务力度，落实领导包保重点项目责任制，完善招商引资机制，兑现招商引资奖惩办法。

　　继续优化和改善发展环境。进一步简化审批程序，清理收费项目，严格落实各项政策。提高政务中心服务水平和办事效率，坚持一门受理、并联审批、封闭运行、限时办结。加强诚信建设。积极帮助企业解决实际困难。严厉查处破坏发展环境的行为，加大问责和处分力度。

　　（六）坚定不移地推进以保障和改善民生为重点的社会建设。坚持以人为本、民生为先，更加注重普通群众意愿，更加突出中低收入群体，更加关注经济调整时期群众的实际困难，保证用于民生的财力只增不减，集中力量为人民群众办一批实事。

　　优先发展教育事业。以办人民满意教育为宗旨，努力解决教育公平问题。积极推进标准化学校工程建设，推动教育资源整合，缩小城乡之间、不同收入群体之间享受教育服务的差距。继续巩固提高义务教育，加快发展高中阶段教育，提高高等教育质量。加强特殊教育、学前教育。大力发展职业教育，全面推进素质教育。建设好技师学院，组建职业高中。加大对农村义务教育投入。完善特困家庭学生救助体系。加强师资队伍建设。

　　全方位抓好就业再就业。密切关注经济形势和就业形势的变化，确保就业形势基本稳定。坚持以创业带动就业，落实小额担保贷款政策，鼓励创业。完善就业援助制度，及时解决零就业家庭就业困难。完善就业服务体系，强化职业技能培训。认真落实政策，鼓励企业吸纳残疾人就业。引导有能力的大学生自主创业和面向基层就业，支持退役士兵自谋职业。扩大劳务输出。强化劳动监察工作，依法维护劳动关系双方合法权益。

　　千方百计增加居民收入。多渠道增加农民收入，严格执行国家粮食最低收购价、农资综合直补、良种补贴、农机具补贴政策，确保农民工工资按时足额发放。建立企业职工工资正常增长和支付保障制度，推动企业建立工资集体协商制度，严格执行最低工资制度。进一步提高企业退休人员基本养老金水平。规范事业单位工作人员津贴补贴，从今年起在全市义务教育学校实施绩效工资。落实好职工带薪休假制度。按时足额发放城乡低保群众、优抚对象的生活补助。

　　着力提高社会保障能力。加快完善社会保障体系，做好社会保险扩面、征缴和发放工作，稳步提高社会保险待遇水平。加大公共财政投入，积极开展农村社会养老保险。完善低收入群体基本生活保障政策，建立健全居民低保与物价变动相适应的动态调整机制。完善优抚保障制度，提高优抚保障水平。加强防灾减灾救灾工作。切实保障残疾人合法权益。支持慈善事业加快发展。

　　加强公共卫生服务。建立健全基本医疗卫生制度，建设覆盖城乡居民的公共卫生医疗服务体系。进一步扩大城镇居民医疗保险覆盖面。加快社区卫生服务机构建设，新建17 个社区服务中心和25 个社区服务站。实施“名医”工程，努力提高医疗技术水平。广泛开展健康知识普及宣传。大力扶持发展中医药事业，改造市中医院。进一步加强人口和计划生育工作，提高出生人口质量。

　　加强保障和改善性住房建设。把保障住房作为政府工作重要任务，完成沉陷区治理工程。改造棚户区60 万平方米。积极探索开发与投资购买相结合的方法，解决廉租房房源问题，新建廉租房1 万平方米，完成实物配租200 套，新增货币补贴家庭1000 户。改造农村泥草房4200 户。完善和谐拆迁配套措施。

　　（七）坚定不移地维护社会安全稳定。越是在经济困难的时候，越要加强稳定工作。坚持安全第一、预防为主、综合治理的方针，深入持久地抓好安全生产。全面落实企业安全生产主体责任和政府安全监管主体责任，以煤矿安全生产为重点，深入开展道路交通、城乡防火、建筑施工、易燃易爆和危险化学品、非煤矿山等重点领域专项整治，严厉打击各种违法违规生产行为，坚决遏制重特大安全事故。加强食品药品质量安全工作，确保群众用药和食品安全。完善公共安全突发事件应急保障和气象灾害防御机制，切实增强应急保障能力。加强平安建设和社会治安综合治理，突出抓好科技强警，构建城区治安防控网络，严厉打击违法犯罪活动，进一步提高人民群众的安全感。

　　下大气力抓好信访工作，落实信访工作责任制，畅通群众诉求渠道，维护人民群众切身利益，引导群众依法表达合理诉求，努力维护社会稳定。

　　（八）坚定不移地加强政府自身建设。坚持以人为本、执政为民，把实现好、维护好、发展好最广大人民群众的根本利益作为出发点和落脚点，努力建设一个行为规范、公正透明、勤政高效、清正廉洁的政府，建设一个让广大人民群众满意的政府。

　　强化发展意识。深入开展学习实践科学发展观活动，认真搞好“查找改”，着力破解思想不解放、发展不够快和工作作风方面的问题。始终保持干事创业的昂扬激情、只争朝夕的顽强干劲，敢闯敢试、敢于创新、敢于突破惯例、敢于担当和负责，殚精竭虑推动发展、造福人民。

　　坚持依法行政。自觉接受市人大及其常委会的依法监督，主动接受市政协的民主监督，定期向市人大及其常委会报告工作，向市政协通报情况、征询意见。进一步加大人大代表和政协委员建议和提案的落实力度。规范行政执法行为，不断提高依法行政能力。

　　推进科学民主决策。坚持问政于民、问需于民、问计于民，实行重大事项集体决策、专家咨询、社会公示与听证等制度，提高政府决策科学化、民主化水平。坚持议大事、抓大事、定大事，在事关发展全局的重大问题上，进一步完善决定重大事项的规则和程序，做到及时、正确决策。

　　全面提高行政效能。着力推动政府职能向创造良好发展环境、提供优质高效服务转变，推动政府管理与服务方式向务实高效、公开透明转变。加强政务服务中心和电子政务建设，推进政务公开。深入开展行政问责和绩效考核，形成雷厉风行、令行禁止的风气，确保政府各项工作落到实处。加强社会管理和公共服务，增强基本公共服务能力，着力解决群众反映强烈的问题。

　　大力加强政风建设。认真落实《建立健全惩治和预防腐败体系2008-2010 年工作规划》，加强政风、行风建设。突出行政监察和审计监督，坚持用制度管权、管事、管人，切实防止以权谋私。严肃查处各类违法违纪案件。厉行节约，降低行政成本。

　　各位代表，我们正处在一个非常之期，把七台河改革发展大业持续推向前进，是摆在我们面前光荣而艰巨的历史任务。让我们高举中国特色社会主义伟大旗帜，更加紧密地团结在以胡锦涛同志为总书记的党中央周围，在市委的领导下，万众一心，攻坚克难，奋力开创我市“358”跳跃式发展的新局面，以优异成绩迎接新中国成立60 周年。
